# Tuberculosis from *Mycobacterium bovis* in Binational Communities, United States

**DOI:** 10.3201/eid1406.071485

**Published:** 2008-06

**Authors:** Timothy C. Rodwell, Marisa Moore, Kathleen S. Moser, Stephanie K. Brodine, Steffanie A. Strathdee

**Affiliations:** * University of California San Diego School of Medicine, La Jolla, California, USA; † County of San Diego Health and Human Services, San Diego, California, USA; ‡ Centers for Disease Control and Prevention, San Diego, California, USA; § San Diego State University, San Diego, California, USA

**Keywords:** Mycobacterium bovis, tuberculosis, trends, incidence, epidemiology, immigration, Mexico, mortality, Hispanic, research

## Abstract

The epidemiology of tuberculosis (TB) in the United States is changing as the incidence of disease becomes more concentrated in foreign-born persons. *Mycobacterium bovis* appears to be contributing substantially to the TB incidence in some binational communities with ties to Mexico. We conducted a retrospective analysis of TB case surveillance data from the San Diego, California, region from 1994 through 2005 to estimate incidence trends, identify correlates of *M. bovis* disease, and evaluate risk factors for deaths during treatment. *M. bovis* accounted for 45% (62/138) of all culture-positive TB cases in children (<15 years of age) and 6% (203/3,153) of adult cases. *M. bovis* incidence increased significantly (p = 0.002) while *M. tuberculosis* incidence declined (p<0.001). Almost all *M. bovis* cases from 2001 through 2005 were in persons of Hispanic ethnicity. Persons with *M. bovis* were 2.55× (p = 0.01) as likely to die during treatment than those with *M. tuberculosis*.

## Abstract 2

The incidence in San Diego is increasing and is concentrated mostly in persons of Mexican origin.

The pattern of tuberculosis (TB) in the United States is changing as the incidence of TB disease becomes more concentrated in foreign-born persons. Of the annual total US TB cases, >54% are now concentrated in persons born outside of the United States (*1*); in communities with high immigration, the proportion can exceed 70% (*2*). TB prevention and treatment strategies, particularly those in communities on the border with Mexico, will need to be adapted to accommodate the changing epidemiology of TB (*3*).

San Diego, California, together with its sister city Tijuana-Tecate, Mexico, is the largest binational metropolitan region in the United States, accounting for 34% of the southern border population (*4*). In San Diego County, the Hispanic population has grown from 20% to 29% in the past 15 years (*5*). Of the total annual TB cases in San Diego, >70% occurred among foreign-born persons, of whom nearly half originated from Mexico (*2*). A review of culture-positive TB cases in San Diego County in the late 1990s indicated that 6.6% of all adult TB cases and 39% of all pediatric (<15 years of age) TB cases from this region were not caused by *Mycobacterium tuberculosis*, the most common TB pathogen in the United States, but were instead caused by *M. bovis*, a pathogen more often associated with TB in cattle (*6*). This finding represented the highest reported proportional incidence of TB from *M. bovis* among industrialized countries (*7*).

*M. bovis* is a pathogen in the complex of bacteria that includes *M. tuberculosis*, which causes TB in humans and animals. TB from *M. bovis* has been generally considered rare in the United States after its successful eradication from cattle in the mid-1900s (*8*), but wider use of laboratory tools for species-level diagnosis of TB pathogens has started to shed light on an unexpected regional presence of *M. bovis* in communities with large Hispanic populations. While *M. bovis* TB has been most often documented in Hispanic communities with close proximity to Mexico (*6*,*9*), a recent review of *M. bovis* cases in New York City indicates that the problem is not limited to US regions that border Mexico (*10*).

The clinical and pathologic characteristics of *M. bovis* TB is indistinguishable from *M. tuberculosis* TB in most cases, but there are relevant considerations for prevention and treatment strategies in communities where *M. bovis* contributes to TB incidence. First, *M. bovis* is thought to be spread to humans primarily through consumption of raw dairy products and inhalation of infectious droplets from cattle (*11*,*12*), with only minimal human-to-human transmission (*13*). Second, *M. bovis* is almost universally resistant to the key antituberculous drug pyrazinamide (PZA), which necessitates a 9-month treatment duration instead of the standard 6-month, short-course therapy, which is possible with PZA in the treatment regime. Third, higher mortality rates during treatment may be associated with *M. bovis* (*14*).

Multidrug-resistant (MDR) strains of *M. bovis* (*15*–*17*), the high proportional incidence of *M. bovis* (*9*,*18*) in pediatric TB cases, and frequent HIV co-infection (*19*) are important additional considerations in developing effective treatment and prevention strategies for *M. bovis*. To document the trends and the effect of *M. bovis* on TB epidemiology, we examined TB case surveillance data from 1994 through 2005 in San Diego County and identified risk factors related to *M. bovis* disease and deaths during treatment in the last 5 years.

## Methods

### Data Sources

This study used routine TB surveillance data from 1994 through 2005. The study protocol was approved by the Institutional Review Boards of San Diego State University and the University of California, San Diego. Demographic and clinical data were obtained from the Tuberculosis Information Management System (TIMS) database maintained by the San Diego County TB Control Program. Since the early 1990s, a TB isolate has been submitted to the county public health laboratory for every reported TB case. All TB isolates from patient specimens were initially identified as *M. tuberculosis* complex on the basis of the AccuProbe hybridization protection assay (GenProbe, San Diego, CA, USA). Specimens were further identified as either *M. bovis* or *M. tuberculosis* on the basis of culture morphologic findings, the results of the niacin strip test, the nitrate reduction test, and the specimens’ susceptibility to PZA (*20*). Furthermore, all isolates identified as *M. bovis* from 2004 and 2005 were confirmed to have spoligotypes consistent with *M. bovis* (*21*). Population data for San Diego County were obtained from San Diego Association of Governments’ estimates based on census and calculated data.

### Study Design

We conducted a retrospective trend analysis of all culture-positive TB cases in the San Diego County TIMS database from 1994 through 2005 that were confirmed as either *M. bovis* or *M. tuberculosis*. We also conducted a detailed retrospective analysis of demographic and clinical variables associated with *M. bovis* case-patients and deaths during treatment from 2001 through 2005.

Demographic variables from the TIMS database used in the correlates and mortality analyses included sex, age, ethnicity, and country of birth. Clinical variables included: previous history of TB disease, presence or absence of pulmonary disease, presence or absence of multisite disease, presence or absence of acid-fast bacilli (AFB) in sputum smear, presence or absence of pulmonary lesions by chest radiograph, presence or absence of MDR TB, and HIV status.

## Analysis

### Trends

Trends in TB incidence were evaluated by using Poisson regression with time in years as the predictor variable, case number as the dependent variable, and population size as an additional exposure variable. Trend lines for *M. bovis* and *M. tuberculosis* were based on incidence predicted by Poisson regression fitted to the data. Trends in proportional incidence of *M. bovis* cases (relative to all TB cases) were assessed with a χ^2^ test for trend.

### Correlates of *M. bovis* Disease

Demographic and clinical variables shown previously to be associated with TB diagnoses (*6*) were compared between *M. bovis* and *M. tuberculosis*. Variables significant at the 5% level by χ^2^ test in univariate analyses were entered into a multiple logistic regression model. The final model was derived by using the likelihood ratio method (*22*).

### Analysis of Mortality Rates during Treatment

All deaths that occurred from the time that a TB case was reported until treatment was completed were documented with death certificates and recorded in TB case files. For the purposes of this study, causes of death in *M. bovis* and *M. tuberculosis* case files were transcribed from death certificates or California state death records and collated into 7 major causes of death based on the most common causes.

We investigated the apparently higher mortality rates during treatment among *M. bovis* cases relative to *M. tuberculosis* cases (*14*) by using a multiple logistic regression analysis with *M. bovis* as the exposure variable; death before treatment was completed as the outcome variable; and demographic and clinical variables as potential covariates. Univariate differences between causes of death in *M. bovis* and *M. tuberculosis* cases were analyzed with the Fisher exact test.

## Results

### Trends

Analysis of TB trends from 1994 through 2005 included 3,291 culture-positive cases of TB and excluded 806 cases (20%) that were based only on national and local clinical case definitions. Among all culture-positive cases, *M. bovis* was isolated in 8% (265/3,291) and *M. tuberculosis* in 92% (3,026/3,291). *M. bovis* accounted for 45% (62/138) of all culture-positive TB cases in children <15 years of age and 6% (203/3,153) of cases in adults (>15 years of age). No cases of *M. bovis* occurred in children <12 months of age.

During the period under study, incident cases of *M. bovis* TB increased linearly (p = 0.002; Figure) at 4.1% per year from 17 cases (0.65/100,000 population) to 28 cases (0.93/100,000) per year. TB cases from *M. tuberculosis* declined in a nonlinear fashion (p<0.001) from 317 cases per year (12.1/100,000 population) to 221 cases (7.33/100,000). The annual proportion of TB cases attributed to *M. bovis* increased from 5% of all culture-positive cases in 1994 to 11% in 2005 (p<0.001, Table 1). The proportion of TB cases with culture-positive results remained relatively stable at ≈81% of annual reported TB cases.

**Figure:**
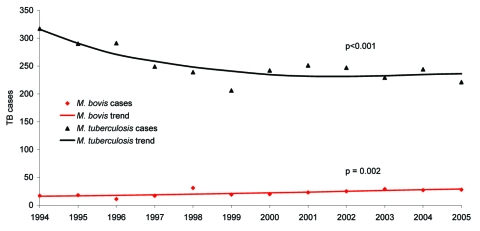
Trends in incidence of culture-positive tuberculosis (TB) cases from *Mycobacterium bovis* and *M. tuberculosis* in San Diego County, California, 1994–2005.

**Table 1:** Proportional contribution of *Mycobacterium bovis* and *M. tuberculosis* to total culture-positive TB cases, San Diego County, California, 1994–2005*

Year	Total TB cases	Total no. (%) culture-positive TB cases†	No. (%)‡ *M. bovis* cases	No. (%)‡ *M. tuberculosis* cases
1994	420	334 (80)	17 (5)	317 (95)
1995	438	308 (70)	18 (6)	290 (94)
1996	384	302 (79)	11 (4)	291 (96)
1997	332	266 (80)	17 (6)	249 (94)
1998	342	270 (79)	31 (11)	239 (89)
1999	299	225 (75)	19 (8)	206 (92)
2000	295	262 (89)	20 (8)	242 (92)
2001	330	274 (83)	23 (8)	251 (92)
2002	317	272 (86)	25 (9)	247 (91)
2003	315	258 (82)	29 (11)	229 (89)
2004	320	271 (85)	27 (10)	244 (90)
2005	305	249 (82)	28 (11)	221 (89)

*TB, tuberculosis. †Excludes 11 case-patients who had an isolate of *M. tuberculosis complex* resistant to pyrazinamide, but did not have species-level identification. Percent given is of all TB cases. ‡Percent given is of total culture-positive TB case-patients.

### Correlates of *M. bovis* Disease

Of the 1,324 culture-positive TB cases reported from 2001 through 2005, *M. bovis* accounted for 10% (132/1,324), comprising 54% (29/54) of cases among children <15 years of age and 8% (103/1,270) among adults (>15 years of age). Of the *M. bovis* TB cases, >96% were found in persons of Hispanic ethnicity, and 60% were among those of known Mexican origin. Univariate analysis indicated that sex, previous TB episode, and sputum AFB smear results were not significantly different between *M. bovis* and *M. tuberculosis* case-patients (Table 2). No MDR TB cases were identified among the *M. bovis* cases, whereas 1.5% of the *M. tuberculosis* cases had initial isolates that were MDR (defined as resistant to at least both isoniazid and rifampin). All of the *M. bovis* isolates were resistant to PZA, whereas 0.8% of the *M. tuberculosis* cases were PZA resistant. Of the 1,316 TB cases included in the multiple logistic regression model, factors associated with *M. bovis* disease included Hispanic ethnicity, multisite disease, being 5–14 years of age, and having extrapulmonary disease with a normal chest radiograph. HIV co-infection was not significantly more associated with *M. bovis* disease compared with *M. tuberculosis* (p = 0.08; Table 3).

**Table 2:** Univariate comparison of *Mycobacterium bovis* cases relative to *M. tuberculosis* cases, San Diego County, California, 2001–2005*

Characteristic	No. (%)†*M. bovis* cases, n = 132	No. (%)† *M. tuberculosis* cases, n = 1,192	Univariate p value
Sex			0.15
M	75 (56.8)	754 (63.2)	
F	57 (43.2)	438 (36.7)	
Age group, y			<0.001
0–4	15 (11.4)	10 (0.8)	
5–14	14 (10.6)	15 (1.3)	
15–24	21 (15.9)	156 (13.1)	
25–44	46 (34.8)	409 (34.3)	
45–64	20 (15.2)	356 (29.9)	
>64	16 (12.1)	246 (20.6)	
Race/ethnicity			<0.001‡
Hispanic	128 (96.9)	529 (44.3)	
White	3 (2.27)	151 (12.6)	
Asian	1 (0.75)	420 (35.2)	
Black	0	86 (7.2)	
Other	0	6 (1.0)	
Country of birth			<0.001
Mexico	79 (59.8)	382 (32.0)	
United States	53 (40.2)	302 (25.3)	
Philippines	0	248 (20.8)	
Other	0	260 (21.8)	
Previous TB			0.52
Yes	5 (3.8)	60 (5.0)	
No	127 (96.2)	1128 (94.6)	
Unknown§	0	4 (0.3)	
Sputum AFB smear result			0.16
Positive	40 (30.3)	590 (49.4)	
Negative	43 (32.5)	461 (38.6)	
Not done§	49 (37.1)	141 (11.8)	
Disease site			<0.001
Pulmonary	71 (53.7)	1031 (86.4)	
Extrapulmonary	61 (46.2)	161 (13.5)	
Clinical manifestations			<0.001
Single site disease	84 (63.6)	1036 (86.9)	
Multisite disease	48 (36.4)	156 (13.1)	
Chest radiograph lesions			<0.001
No lesions	55 (41.7)	123 (10.3)	
Pulmonary lesions consistent with TB	75 (56.8)	1063 (89.2)	
Unknown§	2 (1.5)	6 (0.5)	
HIV status			<0.001
Negative	48 (36.4)	611 (51.3)	
Positive	33 (25.0)	107 (9.0)	
Unknown	51 (38.6)	474 (39.8)	
Treatment outcome			0.02
Died before treatment completed	19 (14.3)	91 (7.6)	
Alive at end of treatment	102 (77.2)	913 (76.5)	
Unknown	11 (8.3)	188 (15.7)	

*TB, tuberculosis; AFB, acid-fast bacillus. †Percentages do not always add up to 100% due to rounding. ‡Fisher exact p value. §Not included in χ^2^ calculation.

**Table 3:** Odds ratios from final logistic regression model of variables correlated with TB  from *Mycobacterium bovis* versus *M. tuberculosis*, San Diego County, California, 2001–2005*

Risk factors	*M. bovis* vs. *M. tuberculosis* (n = 130 vs. n = 1,186)	
	OR (95% CI)	p value
Age group, y (ref >65 y)		0.002
0–4	2.43 (0.81–7.28)	0.11
5–14	4.38 (1.38–13.9)	0.01
15–24	1.06 (0.45–2.49)	0.90
25–44	0.68 (0.31–1.45)	0.32
45–64	0.50 (0.21–1.15)	0.10
Race/ethnicity (ref = white)		<0.001
Hispanic	7.97 (2.36–26.93)	<0.001
Asian	0.08 (0.01–0.76)	0.03
Black	0	0.99
Other	0	0.99
Extrapulmonary disease	4.51 (2.36–8.62)	<0.001
Normal chest radiograph results	3.16 (1.63–6.11)	<0.001
Multisite disease	4.31 (2.54–7.3)	<0.001
HIV status† (ref = negative)		0.13
Positive	1.75 (0.93–3.29)	0.08
Unknown	0.87 (0.48–1.58)	0.65

*n = 1,316. TB, tuberculosis; OR, odds ratio; CI, confidence interval; ref, referent. †Variable not significant in multivariate analysis.

### Analysis of Mortality Rates during Treatment

Of 1,324 culture-positive TB case-patients, 1,119 were evaluated in the analysis of mortality rates during treatment. Fifteen percent (205/1,324) were excluded because of missing data on case survival, including patients who were lost to follow-up or moved during treatment. Of the 1,119 cases, 110 (19 *M. bovis* and 91 *M. tuberculosis*) patients died during TB treatment (n = 81) or before treatment was begun (n = 29). *M. bovis* patients who died during treatment were treated for a similar duration (mean 61 days, standard deviation [SD] 93.9) as *M. tuberculosis* patients (mean 60 days, SD 101.3).

No deaths during treatment were recorded in the pediatric *M. bovis* TB case-patients; 1 pediatric death was recorded among the *M. tuberculosis* TB treatment cases. In a multivariate analysis (n = 1,119), *M. bovis* patients were 2.55× (p = 0.01) as likely to die before treatment completion than *M. tuberculosis* patients, after differences in age, race and ethnicity, country of birth, chest radiograph abnormalities, multisite disease, and HIV status were accounted for (Table 4). Univariate analyses of the causes of death in *M. bovis* and *M. tuberculosis* cases showed no significant differences (p>0.05) except for the category of “other noninfectious disease,” which was overrepresented in the *M. tuberculosis* group (Table 5).

**Table 4:** Factors associated with death before completion of TB treatment in TB patients,  San Diego County, California, 2001–2005*

Risk factor	OR (95% CI)	p value
*Mycobacterium bovis* disease	2.55 (1.27–5.11)	0.01
HIV infection (ref = negative)		<0.001
Positive	4.50 (2.19–9.24)	<0.001
Unknown	2.09 (1.19–3.67)	0.01
Age group, y (ref = 0–4 y)		<0.001
5–14	Not defined	1.00
15–24	0.79 (0.06–9.88)	0.86
25–44	1.54 (0.17–13.78)	0.70
45–64	4.75 (0.55–41.12)	0.16
>65	17.19 (2.04–145.01)	0.01
Country of birth (ref = United States)		<0.001
Mexico	0.52 (0.30–0.90)	0.02
Philippines	0.52 (0.28–0.97)	0.04
Other	0.25 (0.12–0.53)	<0.001
Chest radiograph lesions	2.21 (0.97–5.05)	0.06
Multisite disease	1.86 (1.10–3.15)	0.02

*n = 1,119. TB, tuberculosis; OR, odds ratio; CI, confidence interval; ref, referent.

**Table 5:** Primary causes of death before treatment completion in *Mycobacterium bovis* and *M. tuberculosis* TB case-patients (n = 110), San Diego County, California, 2001–2005*

Cause of death	No. (%) *Mycobacterium bovis* case-patients	No. (%) *M. tuberculosis* case-patients	p value†
Pulmonary TB	2 (11)	20 (22)	0.42
Extrapulmonary TB	3 (16)	8 (9)	0.58
Pulmonary NOS	0	8 (9)	0.41
HIV related	6 (32)	12 (13)	0.11
Cardiovascular	7(37)	18 (20)	0.19
Infectious (not TB)	1(5)	2 (2)	0.84
Other noninfectious disease	0	23 (25)	0.01
Total	19	91	

*TB, tuberculosis; NOS, not otherwise specified. †Fisher exact test.

## Discussion

From 1994 through 2005, incidence of *M. bovis* TB cases in San Diego County increased in absolute number, as a proportion of total TB cases, and relative to the population. In contrast, TB incidence caused by *M. tuberculosis* declined during the same period. *M. bovis* cases were concentrated in persons of Hispanic descent, especially those of Mexican origin, and among those <15 years of age, in whom *M. bovis* accounted for 45% of the culture-positive cases. Deaths during treatment were largely confined to adults and were twice as high in *M. bovis* TB case-patients when compared with *M. tuberculosis* patients.

Our findings indicate that the incidence of TB caused by *M. bovis* in southern California is substantially higher than the national rate of 1.5% estimated from TB surveillance data (*23*) but is similar to the proportional incidence (13%) among Mexican-born case-patients in New York, New York. It was previously hypothesized that TB attributed to *M. bovis* in San Diego is most likely being driven by recent infections in children and largely reactivated latent infections in adults, secondary to HIV co-infection (*6*). Our findings confirm the continued high incidence of *M. bovis* in children >12 months of age, but the role of HIV co-infection in *M. bovis* case-patients relative to *M. tuberculosis* cases is less clear.

Almost half of the culture-positive pediatric TB cases in this binational region of >3 million persons were caused by *M. bovis*, which has clinical implications. Since *M. bovis* is intrinsically resistant to PZA, a critical component of the standard 6-month, short-course treatment for *M. tuberculosis*, *M. bovis* treatment is usually extended to 9 months of isoniazid and rifampin (*14*). In southern California, and perhaps other Hispanic communities with close ties to Mexico, empiric extended TB treatment for children without culture-positive disease, particularly those with a history of consuming unpasteurized dairy products, should be considered.

Although one quarter of the *M. bovis* TB case-patients were co-infected with HIV, HIV was not significantly more associated with *M. bovis* TB compared with *M. tuberculosis* in our study or in the previous *M. bovis* study in this community (*6*). Confidence in this finding is somewhat limited because 40% of the TB case-patients did not have their HIV status reported. However, because most of the case-patients with an unknown HIV status were <15 or >55 years of age, the age groups at lowest risk for HIV, these missing data likely did not mask an association if one exists.

HIV co-infection plays a role in the epidemiology of adult *M. bovis* TB, but likely the growing Hispanic population with close ties to Mexico, and not HIV, is the major driving force behind the increasing number of *M. bovis* cases we observed. Given the long latency of this disease, the unclear role of airborne transmission in *M. bovis* TB (*13*), and the fluid population dynamics of the San Diego region, however, the true population at risk and what might be behind the trends observed are difficult to describe. The relative contributions of reactivated latent *M. bovis* infection compared with recent infection could not be discerned in our study, but prudent prevention strategies would include a focus on eliminating consumption of unpasteurized dairy products in both adults and children in the United States and Mexico.

Our study confirms earlier preliminary findings (*14*) that *M. bovis* case-patients appear to be more than twice as likely to die before TB treatment completion compared with *M. tuberculosis* patients, despite being treated for the same mean number of days. The association of higher mortality rates during *M. bovis* treatment persisted after HIV, multisite disease, age, and ethnicity were accounted for. Causes of death related to noninfectious disease, such as malignancy and noninfectious gastrointestinal pathologies, were underrepresented in the *M. bovis* cases (0 vs. 25%), but, overall, the *M. bovis* and *M. tuberculosis* cases were not significantly different with regard to all causes of death.

In mouse models, evidence indicates that certain strains of *M. bovis* are more virulent than *M. tuberculosis* strains (*24*), but those findings are not generally supported in the literature on human *M. bovis* TB (*7*). Although our mortality analysis partially controlled for extent of disease, it did not include information on coexisting conditions, stage of HIV disease, diagnostic delays, and prior access to medical care. Therefore, *M. bovis* deaths might be accounted for by other factors, such as health disparities or treatment differences, which warrant further investigation.

Public health measures to control TB are currently focused on interrupting person-to-person transmission by promptly identifying and treating infectious patients and ensuring that they do not expose new contacts until treatment has rendered them noninfectious. Based on our data, these strategies, which have proven to be effective at reducing *M. tuberculosis* cases in San Diego and most regions of the United States, appear to be less effective in controlling *M. bovis*, suggesting that human-to-human transmission of *M. bovis* is less likely an important mode of transmission in this community. The consumption of contaminated dairy products has been proposed to be the primary source of human TB from *M. bovis* (*25*). This hypothese is supported by the findings of an investigation of *M. bovis* cases in New York that indicated the likely source of infection was unpasteurized cheese from Mexico (*10*). Additionally, San Diego pediatric *M. bovis* cases occur only after the age of weaning, when children are typically first exposed to dairy products (*6*), and *M. bovis* was also recently cultured from unpasteurized cheese seized at the San Diego–Mexico border (*26*,*27*).

Because of the widespread adoption of pasteurization of all commercially available dairy products in the United States, as well as the aggressive US state agricultural health programs designed to keep dairy cattle free from *M. bovis* disease, the threat of *M. bovis* in US dairy products was largely eliminated in the mid-20th century (*8*). The San Diego–Tijuana binational region, however, shares one of the busiest border crossings in the United States with Baja, Mexico (*28*), where *M. bovis* is prevalent in cattle and consumption of unpasteurized dairy products is a common cultural practice (*29*–*32*). Mexican dairy products, including the popular *queso fresco* (soft, unpasteurized cheese), may be brought into the United States for personal use and are sometimes distributed illegally (*27*). Given our finding that >90% of *M. bovis* cases in San Diego occurred in Hispanics, most of whom were born in Mexico, consumption of unpasteurized dairy products from Mexico is likely a major risk factor for *M. bovis* TB in San Diego. Collaboration with Mexico on prevention strategies, from education to regulation of the production of unpasteurized dairy products, and elimination of *M. bovis* from dairy cattle will be required in the long term to ensure that this mode of transmission is eliminated.

### Limitations

A growing awareness of *M. bovis* as a cause of TB in San Diego since 1980 could have introduced a sampling bias into our trend estimates, but this possible bias is unlikely to have had a considerable effect in the years 1994 through 2005 as reported here. All suspected TB cases in San Diego County are reportable to the health department, and the county laboratory has consistently conducted testing to distinguish all *M. tuberculosis* complex isolates as either *M. bovis* or *M. tuberculosis* since 1994. The proportion of TB cases based only on clinical diagnosis and not species level culture has remained relatively level, at ≈20% of all reported TB cases. Increased efforts to obtain specimens for culture in pediatric TB cases in the years under study did not appear to change the proportion of culture-positive cases during the study period.

The cohort of TB case-patients who were not culture-positive and thus excluded from this analysis was significantly different from the study group. The <15-year age group (36% vs. 4%, respectively) and Hispanic ethnicity (57% vs. 45%) were both overrepresented in the excluded cases. Given that these are the groups most likely to have *M. bovis* TB, the total incidence of *M. bovis*, particularly in children, may be underestimated in our study.

## Conclusions

San Diego, California, while unique in many respects because of its close proximity to Mexico, is possibly representative of other communities in the United States with large and growing Hispanic populations with ties to Mexico. The considerable and growing incidence of TB from *M. bovis*, especially in children, and the observed number of deaths during treatment in these cases is of serious concern. It raises the question of the importance of incorporating routine species-level identification into US TB surveillance as the national TB incidence shifts to persons born outside the United States. This surveillance will be greatly facilitated by the national genotyping project implemented by the US Centers for Disease Control and Prevention in 2004 (*33*), and its use will be particularly important for communities with strong ties to Mexico.
